# Ventilators and Ventilatory Modalities

**DOI:** 10.3389/fped.2020.00500

**Published:** 2020-09-02

**Authors:** Martino Pavone, Elisabetta Verrillo, Alessandro Onofri, Serena Caggiano, Renato Cutrera

**Affiliations:** Pediatric Pulmonology & Respiratory Intermediate Care Unit, Sleep and Long Term Ventilation Unit, Academic Department of Pediatrics (DPUO), Pediatric Hospital “Bambino Gesù Research Institute, Rome, Italy

**Keywords:** ventilators, non-invasive ventilation, pressure support ventilation, pressure control ventilation, hybrid modes

## Abstract

Non-invasive ventilation is increasingly used in children for acute and chronic respiratory failure. Ventilators available for clinical use have different levels of complexity, and clinicians need to know in detail their characteristics, setting variables, and performances. A wide range of ventilators are currently used in non-invasive ventilation including bi-level ventilators, intermediate ventilators, and critical care ventilators. Simple or advanced continuous positive airway pressure devices are also available. Differences between ventilators may have implications on the development of asynchronies and air leaks and may be associated with discomfort and poor patient tolerance. Although pressure-targeted (controlled) mode is preferable in children because of barotrauma concerns, volume-targeted (controlled) ventilators are also available. Pressure support ventilation represents the most used non-invasive ventilation mode, as it is more physiological. The newest ventilators allow the clinicians to use the hybrid modes that combine the advantages of volume- and pressure-targeted (controlled) ventilation while limiting their drawbacks. The use of in-built software may help clinicians to optimize the ventilator setting as well as to objectively monitor patient adherence to the treatment. The present review aims to help the clinician with the choice of the ventilator and its ventilation modalities to ensure a successful non-invasive ventilation program.

## Introduction

Non-invasive ventilation (NIV) is increasingly used in children for acute and chronic respiratory failure (1, 2).

Ventilators available for clinical use have different levels of complexity and clinicians need to know their characteristics, setting variables, and performances (3, 4).

Differences between ventilators can have implications on air leaks and patient–ventilator interaction, causing asynchronies that may lead to poor NIV tolerance (5).

If the clinician fails to optimize the setting to respond effectively to the patient's requests, there is a greater probability of NIV failure. For these reasons, the choice of the ventilator and the clinician proficiency are crucial steps to improve the chance of a successful NIV program (3–5).

This review is part of the research topic “Pediatric Long-Term Non-Invasive Ventilation,” indications and modes of NIV in children affected by chronic respiratory failure will be extensively discussed in other articles that are part of this research topic.

## Types of Ventilator

There is no consensus about the classification of ventilators; however, they can be categorized as bi-level ventilators, intermediate ventilators, and critical care ventilators (3, 4).

For proper functioning, all ventilators require electricity. This can be supplied as external alternating current or via an internal direct current battery.

Usually, the gas source can be a piston, micro-piston, or more recently a turbine. Fast turbines (also known as dynamic blower systems), or turbines rotating at constant speed (also known as constant-revolution blower systems) are highly efficient and are used in the latest generation of turbine-driven ventilators (6).

Most ventilators only possess a low-pressure oxygen inlet. With this configuration, oxygen delivery is not constant and very high FiO_2_ levels cannot be achieved. Some ventilators may work with oxygen at high pressure and may deliver a constant preset oxygen tension, independent of the minute volume ventilation (7).

NIV in children can be performed using volume-targeted or pressure-targeted (controlled) ventilators, based on the *control* variable with which the ventilator delivers the inspiration (4).

A ventilator produces a volume-targeted or a pressure-targeted ventilation if, respectively, the wave profile of the flow (in volume-controlled mode) or pressure (in pressure-controlled mode) delivered during inspiration does not vary with changes in the mechanics of respiratory system in terms of compliance and resistance (4).

In volume-targeted ventilation, the mechanical breath generates a preset volume during a given time. In pressure-targeted ventilation, the mechanical breath generates a preset pressure during a given time (4). Some ventilators are able to combine volume and pressure, so that hybrid modes can be performed (8).

In NIV, pressure-targeted (controlled) mode is the most often used due to its ability to compensate for leaks and to generate constant pressures when compared with volume-targeted ventilation (8, 9).

This article will focus mainly on positive pressure ventilation modes in the long-term setting. There will be a section dedicated to hybrid modes and a summary on volume-targeted ventilation. Critical care ventilators used in the acute clinical setting will not be described.

### Bi-Level Ventilators

Bi-level ventilators are the most common devices used to perform NIV. These ventilators are configured with a single-limbed vented circuit and designed to operate in the presence of leaks (8).

The leaks are necessary to flush the circuit of exhaled air gases and carbon dioxide through a leak port. These ports can be located either in the circuit (i.e., whisper swivel, Philips Respironics, Murrysville, PA, USA) very close to the patient interface or in the interface (i.e., so-called vented masks). The risk of rebreathing is reduced by using a baseline expiratory positive pressure. In some bi-level ventilators, expiratory pressure cannot be set below 3 or 4 cmH_2_O to assure that the circuit is adequately flushed (4, 8, 9).

The newest bi-level ventilators allow the clinician to indicate the interface used during NIV (9). This feature allows the device to better identify intentional and unintentional leaks so that the flow can be adjusted to guarantee desired performances. Estimates of tidal volume and leaks are determined through algorithms specific to the devices (8). Bi-level ventilators work properly in the presence of certain amount of leaks that may vary between devices. Leaks that impair proper trigger or cycle functioning must be considered as unacceptable (9).

The newest bi-level ventilators provide several modes, displays, alarms, batteries, and internal oxygen blenders (3, 4, 6–8).

### Intermediate Ventilators

Intermediate ventilators may have single- or dual-limbed circuit configurations. Single-limb configuration has either an active exhalation valve usually close to the patient airway (non-vented configuration) or a passive leak (vented configuration). Current intermediate ventilators allow various pressure- or volume-targeted modes. Some devices may also be equipped with hybrid modes like average volume-assured pressure support and intelligent volume-assured pressure support (8).

Like bi-level devices, intermediate ventilators possess several modes, displays, graphics, alarms, batteries, and internal oxygen blenders (3, 4, 6–8).

## Respiratory Circuits

Ventilators deliver pressure to the airway by a respiratory circuit. The proximal part of the respiratory circuit is connected to the ventilator, and the distal part is connected to the patient through a non-invasive interface.

As briefly mentioned above, three main types of circuit are available.

In the single-limb respiratory circuit, inspiration and expiration occur through the same limb, and this would potentially lead to carbon dioxide rebreathing (10). In order to avoid this rebreathing, two different systems are available:

1) The *non-vented* respiratory circuit is a single circuit equipped with a non-rebreathing expiratory valve. This valve (e.g., a mushroom valve driven by ventilator pressure) has an on–off function and allows complete elimination of carbon dioxide. Usually, this circuit provides only inspiratory tidal volume measurement (11).
2) The *vented* respiratory circuit or intentional leak respiratory circuit is a single respiratory circuit without a “true” non-rebreathing active valve. In this configuration, carbon dioxide is vented out through passive exhalation ports (i.e., whisper swivel, Philips Respironics, Murrysville, PA, USA). The efficacy of this exhalation may be affected by several factors such as the level of expiratory positive airway pressure, the combined amount of intentional and unintentional leaks, and the supplemental oxygen delivered into the non-invasive interface. In this configuration, inspiratory or expiratory tidal volumes are indirectly calculated by an algorithm.

The double-limb respiratory circuit includes an inspiratory and expiratory limb. The proximal parts of the respiratory circuits are connected, respectively, to the inspiratory and expiratory ports of the ventilator where inspiratory and expiratory valves are positioned. The distal parts of the respiratory circuits are connected to the Y-piece ending in the patient interface (10). This circuit configuration allows one to measure inspiratory and expiratory tidal volume.

## NIV Terminology

Terminology associated with NIV modes may vary between bi-level, intermediate, and critical care ventilators, and this may generate confusion. On devices designed primarily for NIV (i.e., bi-level ventilators), the pressure setting is indicated as inspiratory positive airway pressure (IPAP) during inspiration and as expiratory positive airway pressure (EPAP) during expiration. On other devices, including critical care ventilators, during inspiration, the effective pressure generated is the sum of the inspiratory assistance added to the preset positive end expiratory pressure (PEEP) (4, 9).

In the context of the delivery of a positive pressure breath, several variables need to be defined. The *trigger* defines the onset of inspiration criteria, the *limit* defines the inspiratory limit that cannot be overcome during inspiration, the *cycling* defines the transition from inspiration to expiration criteria (3), and the *control* defines whether a breath is volume or pressure controlled.

A mechanical breath can be *triggered* by pressure; volume; a combination of pressure, flow, and volume; waveform algorithms; or time (3, 4, 9). Pressure and flow triggering, respectively, allows detection of a pressure drop or a flow modification within the circuit determined by the patient's inspiratory effort. Newer devices improve patient–ventilator synchrony trough algorithm that combines volume, pressure triggers, and a flow waveform algorithm. Many ventilators enable the clinician to set the triggers' threshold *sensitivity*.

The main goals of a performing inspiratory trigger are the reduction of both the intensity of the muscular effort and the delay between patient's inspiration onset and starting a ventilator delivered breath.

*Limit* variables are the minimal or maximal values manually adjustable for a mechanical breath according to the set options available among the device features (i.e., minimum inspiratory time, *T*_min_, and maximum inspiratory time, *T*_max_, in a flow cycled breath).

A mechanical breath can be *cycled* by pressure, time, volume, or flow (3). A breath is defined as time-cycled when it is terminated after a given preset inspiratory time. A breath is defined as flow-cycled when it is terminated after a given inspiratory flow threshold decay (3, 4, 9). Many ventilators allow the clinician to set the *sensitivity* of the threshold of flow termination criteria. This threshold is usually expressed in terms of percentage of peak inspiratory flow or labeled as number.

## Ventilation Modes

Ventilation should be considered as a double compartment respiratory model, where expiratory pressure will favor the upper compartment (upper airway) patency needed to allow the delivery of pressure support to the lower compartment (lower airways) (12).

Non-invasive intermittent positive pressure (NPPV) ventilation differs from continuous positive airway pressure (CPAP) because it provides two different levels of pressure. During NIV, pressure increases during the inspiratory phase of the breath and returns to an elevated baseline during expiratory phase. The pressure increases during the inspiratory phase, augments tidal volume, improves gas exchanges, and unloads respiratory muscles (3, 4, 9).

### Continuous Positive Airway Pressure

CPAP is a spontaneous modality in which the work of breathing is completely generated by the patient. Since CPAP does not provide pressure assist during inspiration, it should not be considered a “true” ventilation mode (4). CPAP is usually described in the context of NIV because it shares the circuits, interfaces, and sometimes the same ventilators.

CPAP is based on the delivery of fixed preset pressure to the airways for the entire respiratory cycle (3, 4, 9, 12). On the upper airways, working as mechanical stent, CPAP increases their cross-sectional area and keeps the airway open by elevating the intraluminal pressure above the transmural critical pressure that determines the collapse. On the lower airways, releasing a continuous additive flow, CPAP may prevent alveolar collapse, favors the alveolar recruitment, and increases the functional residual capacity.

Through these mechanisms, CPAP counteracts the obstruction of the upper airways, may prevent atelectasis, improves oxygenation, and downloads the inspiratory muscles, reducing the work of breathing. Moreover, CPAP may reduce afterload and increase cardiac output by lowering left ventricular transmural pressure (3, 4, 9, 12). CPAP may also stabilize chest wall distortion.

Auto-titrating CPAP is an advanced mode during which the delivery of positive airway pressure is adjusted automatically between a range of values set by the clinician, according to either the analysis of the flow curve or airway resistance [forced oscillation technique (FOT)] performed by the device's software. By using auto-titrating CPAP devices, pressures may vary based on the patient's needs at various time of the night (i.e., higher pressure during REM sleep). This could allow the patient to receive, on average, lower pressures during the night and experience fewer side effects related to higher pressures (13).

In some devices, more advanced CPAP modes are also available. These modes are mainly characterized by a moderate decrease in airway pressure at the beginning of expiration, or a variable increase in airway pressure during inspiration (14).

Manufacturers recommend a minimum weight (10–30 kg), for use of the auto-titrating and advanced CPAP modes.

### Volume-Targeted (Controlled) Ventilation

In volume-targeted (controlled) ventilation, as previously mentioned, the ventilator is set to deliver a fixed volume (independent variable) during a given time span. This fixed volume will be delivered whatever pressure (dependent variable) is necessary to reach the target, regardless of the patient contribution to ventilation. The effective pressure released to the airways will depend on the interaction between ventilator settings, spontaneous patient inspiratory efforts, and mechanics of the respiratory system. Further inspiratory effort does not change delivered volume or flow (9, 15, 16).

The advantage of this mode is the strict delivery of the preset volume in the absence of leaks and regardless of the compliance and resistance of the respiratory system. The disadvantages of this mode are essentially two. The first is the delivery of a fixed volume that occurs independently of the varying needs of the patients. The second is that, with increasing leaks, there is no proportional compensatory increase in flow rate, which results in lower effective pressure and reduced volume (9, 15, 16).

### Pressure-Targeted (Controlled) Ventilation

In pressure-targeted (controlled) ventilation mode, the ventilator delivers airflow by generating a preset positive pressure (independent variable) in the airways for a given time (9, 15). Flow is the dependent variable. Thus, the volume delivered in the airways will not be fixed and will result from the interaction between the patient inspiratory effort, the preset pressure, the inspiratory time, and the mechanics of the respiratory system (15).

Advantages of pressure-targeted (controlled) ventilation include the ability to compensate for mild to moderate leaks and improvements in synchronization since flow can vary breath by breath (3, 4, 9, 15) A limitation of pressure-targeted (controlled) ventilation mode is that tidal volume cannot be guaranteed, and this may potentially lead to insufficient ventilation (9, 15). Bi-level positive airway pressure (bi-level) provides respiratory support at two different levels (4, 9, 15, 16). Bi-level ventilation allows, therefore, the clinician to set independently an expiratory and an inspiratory positive airway pressure.

Similarly to CPAP, adding an expiratory positive airway pressure helps keep the upper airways open, and through alveolar recruitment, it reduces the risk of atelectasis and favors the increase of functional residual capacity.

Moreover, in a single limb circuit with a passive exhalation port or a vented interface, expiratory positive airway pressure would prevent the re-breathing of carbon dioxide (10, 11).

The tidal volume will result from the difference between inspiratory and expiratory pressures within certain limits, the flow resistance of the respiratory circuit, any airflow limitations, and the mechanics of the respiratory system (4, 15).

Based on the patient–ventilator interaction, bi-level PAP can be delivered in PSV mode [either in spontaneous (S) or spontaneous timed (ST) mode], or in pressure-controlled ventilation (PCV) mode. The devices may also include a timed (T) or control mode, rarely used because it does not allow for patient synchrony.

#### PSV Mode

PSV is a pressure-controlled flow-cycled mode. In the *spontaneous* (*S*) mode, in bi-level ventilators, inspiration starts when the patient triggers the ventilator. The inspiratory pressure is maintained as long as a minimum preset inspiratory flow is present. The switch from inspiration to expiration (cycling) occurs when inspiratory flow achieves a preset percentage of peak inspiratory flow. Therefore, in this mode, the patient controls the onset (triggering) and end (cycling) of inspiration, the ventilator supports the respiratory act, and again the patient determines the respiratory rate and pattern (4, 17).

Based on the ventilator used, in spontaneous mode, the clinician can select a target inspiratory positive pressure, trigger sensitivity, and the threshold of peak flow for cycling to expiration. Usually, this threshold is set at 25% of inspiratory peak flow, but most of the available ventilators allow the setting of a wide range of threshold (i.e., 5–80%) (11). Some simpler ventilators allow the clinicians to set only the inspiratory positive pressure (15, 17).

In the *spontaneous/timed* (*ST*) mode, a combination of spontaneous supported flow cycled breaths and mandatory mechanical acts is allowed.

If the patient's spontaneous respiratory rate is lower than the backup respiratory rate, mechanical breaths are triggered, supported, and cycled by the ventilator (4, 17).

During patient-triggered breath, the ventilator cycles in expiration when it senses a drop in inspiratory flow rate below a preset threshold. During device-triggered breath, the ventilator cycles in expiration at a preset time (17).

Based on devices, in this mode, the clinician determines inspiratory and expiratory pressures, backup respiratory rate, and rise (pressurization) time (16). Some devices allow the clinician to dial an inspiratory time, while others allow to dial a range from a minimum (*T*_min_) to a maximum inspiratory time (*T*_max_) (12).

Bi-level ventilators adopt the terminology IPAP for inspiratory pressure and EPAP for expiratory pressure. While PSV works above a given PEEP level, in bi-level ventilators, if EPAP needs to be increased, the IPAP should be increased as well to keep the same level of inspiratory support. This adjustment maintains the difference between inspiratory and expiratory pressures (4).

A flow cycled breath allows to preserve the patient's spontaneous breathing and to reduce excessive work of breathing (4, 9, 12, 17). PSV is advisable in any condition in which the patient's spontaneous breathing can sustain the proper minute ventilation. On the contrary, this mode is not recommended in patients with significant impairment of the ventilatory drive, with severe depression of consciousness or severe deterioration of muscle pump efficiency (18).

#### PCV Mode

PCV is a pressure-controlled time-cycled mode. In the *assist* (*A*) mode, inspiration starts when the patient triggers the ventilator. The inspiratory pressure lasts for a preset time (16). Cycling from inspiration to expiration occurs after a given set time (15, 17). Therefore, the patient controls the beginning (triggering) of inspiration, but the inspiratory pressure level, inspiratory time, and cycling to expiration are provided mechanically by the ventilator. In assist mode, the respiratory rate will be determined by the patient, but the respiratory pattern will be determined by the ventilator.

In the *control* (*C*) mode, the ventilator controls the beginning of inspiration (time triggering), the end of inspiration (cycling), and the respiratory rate (17). In this mode, the ventilator performs the entire work of breathing. Some ventilators call this Timed (T) mode (17).

When a backup respiratory rate is applied, this mode is defined as pressure assist/control (AC-PCV) mode (4, 9, 17). In this last mode, a combination of assisted spontaneous breathing and controlled acts is allowed. If the patient's spontaneous respiratory rate is lower than the preset ventilator backup respiratory rate, the system switches from assist (patient-triggered breaths) to control (device-triggered breaths) mode. Thus, triggering by the patient is allowed, but the ventilator delivers a breath with the same inspiratory time of the mandatory breath.

Based on devices, in AC-PCV mode, the clinician selects inspiratory and expiratory pressures, inspiratory: expiratory (I:E) ratio, or inspiratory time, inspiratory trigger sensitivity, and rise time (17). However, inspiratory time in patients with a respiratory activity should be set according to the actual patient's rate. For this reason, many clinicians prefer the ST mode.

This mode is advisable in severely ill patients with significant impairment of the ventilatory drive or of the muscle pump efficiency (18).

## Hybrid Modes

As previously briefly discussed, some new ventilators provide hybrid modes. Hybrid modes, known as volume-targeted (adaptive) pressure ventilation, use intelligent algorithms to automatically adjust the setting to achieve predefined targets (9, 17). Hybrid modes combine the advantages of conventional volume and pressure-targeted (controlled) ventilation (8, 17) and can be used in either pressure support or control mode.

Ventilators may have different algorithms and setup variables, and this explains differences in their response (8, 9, 15, 17). Some ventilators can adjust a target volume within each cycle, while others can progressively adjust the pressure level during several cycles (8, 17).

Ventilators can provide volume-targeted (adaptive) pressure ventilation with all the respiratory circuit configuration previously described (9, 16).

Volume-targeted (adaptive) pressure ventilation is, essentially, an adaptive dual-targeting mode that should permit the ventilator to properly compensate for possible changes in respiratory mechanics ensuring a constant and effective ventilation (8). According to the different algorithms, adjustments in inspiratory pressure (first target) take place to deliver the predetermined target volume (second target) (8).

The control variable is inspiratory pressure, constrained between a range of values (minimum and maximum) set by the clinician. Inspiration starts as a pressure-targeted (controlled) mode. Once the devices have measured or estimated the delivered volume, it determines whether to remain unchanged or to modify, before cycling to expiration, the inspiratory pressure level to achieve the dependent variable, which is the preset target volume (8, 16, 18).

For a given patient, the minimum inspiratory support should be set to a safe level of tidal volume. The maximum pressure support should be set to allow the ventilator to increase inspiratory pressures and compensate for drops in target volume due to air leaks or reduced inspiratory effort (8).

In addition to the range of inspiratory pressures, the settings include the titration of the expiratory positive airway pressure with the aim to maintain airway patency. The expiratory positive airway pressure can be fixed or, in some ventilators, automatically adjusted between a range of pressures (minimum and maximum) set by the clinician. To achieve this goal, most new ventilators use a combination of snore and flow detection (8, 12).

Moreover, some new ventilators allow setting a variable backup respiratory rate. By automatically adjusting inspiratory and expiratory pressures and backup respiratory rate in a preset range to achieve a target ventilation, these ventilators are able to provide a fully automatic mode (8, 12, 17).

Some ventilators include a learning mode in which the device tends to reproduce the patient's breathing pattern and determines target ventilation (8, 9). However, the use of this mode is not standard practice.

### Average Volume-Assured Pressure Support

Average volume-assured pressure support is a form of volume-targeted (adaptive) pressure control ventilation in which the level of pressure support adapts to deliver an average tidal volume (14). In this mode, the target is the expiratory tidal volume and the tidal volume produced by the patient is averaged over 1 min. Then, the algorithm changes the inspiratory pressure according to the speed rate set by the clinicians (from ± 1 up to 5 cm H_2_O per minute) for the subsequent breaths until the target tidal volume is reached (12).

The target tidal volume can be determined through various methods including those based on ideal body weight, measurement of carbon dioxide level during wakefulness or sleep, or by determining a “comfortable” level for that patient and then setting the goal 110% higher.

Some devices possess the auto-EPAP (AE) algorithm. In the average volume-assured pressure support-AE devices, the technique of the forced oscillations is used to measure the airway resistance. In the presence of obstructed airways, the flow oscillations of the forced oscillation technique sinusoidal signal will be smaller than a baseline with patent airways and the EPAP will increase, within preset limits, after the analysis of several breaths (12).

In average volume-assured pressure support mode, backup respiratory rate can be fixed or autoset (two breaths less than the average rate of the most recent six resting spontaneous breaths).

Usually, in this mode, the clinician sets tidal volume, minimum/maximum inspiratory pressures, expiratory pressure (or minimum/maximum expiratory pressure in the case of the auto-EPAP), backup respiratory rate, and rise time (14).

Accurate monitoring of the actual tidal volume is crucial for proper algorithm compensation. Expiratory tidal volume can be measured in the presence of a pneumotacograph placed on the expiratory port of the ventilator as for some turbine-driven ventilators configured with a double-limbed circuits. In case of leaks, this configuration may underestimate the real tidal volume and overcalculate the delivered volume (19). Expiratory tidal volume can be estimated in the absence of a pneumotacograph as for single limb intentional-leak circuit configuration. Some ventilators, even during constant leakage, are able to accurately estimate expiratory tidal volume, rebuilding the patient's flow pattern considering the ventilator's turbine speed, the detection of leaks (either intentional or unintentional), and the onset and the end of inspiration (19). In this way, the ventilators calculate a baseline breathing pattern (patient's zero flow) to obtain an estimated expiratory tidal volume equal to the inspiratory tidal volume (8, 20).

This mode is increasingly used for the management of children poorly responsive to the previously described modes. Successful experiences have been described in infants, children, and adolescents with neuromuscular disease (congenital myopathy) (21), disorders of ventilatory drive (congenital central hypoventilation syndrome) (22–24), and morbid obesity (25).

### Intelligent Volume-Assured Pressure Support

Intelligent Volume-Assured Pressure Support is designed to maintain a predefined target alveolar minute ventilation. This target is achieved by monitoring the delivered ventilation, adjusting the inspiratory pressures and automatically activating an intelligent backup respiratory rate (8, 9, 12). The intelligent volume-assured pressure support is indicated for patients weighing 30 kg or more.

The pressure support is continuously adjusted breath by breath in order to maintain target alveolar ventilation. The pressure support adjustment range is limited by the values of minimum and maximum pressure. The breath-by-breath changes in pressure support depend on the respiratory rate and the difference between actual and target alveolar ventilation (12).

The alveolar ventilation value is obtained by subtracting the estimated dead space from a minute ventilation target. Either dead space or minute ventilation can be estimated by indicating the patient's height and respiratory rate, or by selecting disease specific preset values (for normal, obstructive, restrictive lung mechanics, and obesity hypoventilation) available within the device features. The clinician can manually increase or decrease the programmed target alveolar ventilation (12). The target alveolar ventilation can also be determined by measuring of carbon dioxide level during wakefulness or sleep, or assessing the patient comfort at a particular setting (8, 9, 12).

The intelligent backup rate self-regulates the respiratory rate between two limits. The upper limit for intelligent backup rate is the patient's target respiratory rate and should be set to match the patient's average spontaneous rate. The lower limit for intelligent backup rate is two-thirds of the patient's target respiratory rate. During spontaneous breathing, the intelligent backup rate adjusts to two-thirds of the patient's target respiratory rate in order to let the patient spontaneously activate the inspiratory trigger. When the spontaneous inspiratory trigger ceases (e.g., at the beginning of an apnea/hypopnea), the intelligent backup rate intervenes by delivering the patient's target respiratory rate. A single breath with spontaneous inspiratory trigger returns the intelligent backup rate to two-thirds of the patient's target rate (15).

The cycling variables can be either inspiratory time or the percentage of inspiratory flow decay.

The expiratory positive airway pressure can be fixed (manually set) or automatically adjusted within a range of pressure values (minimum and maximum) set by the clinician (8).

In addition, intelligent volume-assured pressure support allows a “learning” mode, which is a period of time (usually 20 min during spontaneous breathing under 4 cmH_2_O) during which the device software, by measuring the patient's respiratory rate and tidal volume, computes a target minute ventilation (12, 15).

There is currently very little data in the literature on the use of this ventilation mode in children. A study performed on children with congenital central hypoventilation syndrome reports that the use of the intelligent volume-assured pressure support was associated with a reduction in the maximum transcutaneous carbon-dioxide level during NREM sleep as compared to traditional ST mode (26).

## Asynchronies

The superimposition of mechanical breaths on spontaneous breathing children remains a challenge for a number of reasons including the age-related small tidal volumes and the high respiratory rates (5, 27). These factors combined with the air leaks may lead to patient–ventilator asynchrony (27).

A patient–ventilator asynchrony occurs when one or more phases of breath delivered by the ventilator do not match the phases of breath of the patient (5, 27, 28).

There are various classifications of the patient–ventilator asynchrony and there is no definitive consensus (5, 27–29). They can be classified as asynchronies occurring during the inspiratory period, during the transition from inspiration to expiration, and during the expiratory period.

Below are reported the most frequent patient–ventilator asynchronies. For further details, see specific articles on this topic (5, 27, 28).

*Ineffective triggering* (also called *missed triggering* or *wasted effort*) occurs when an inspiratory muscle effort is not followed by a ventilator mechanical breath. This type of asynchrony can occurs when the patient initiates a breath that does not reach the ventilator's trigger threshold (5, 27, 28).

There are some situations (called *trigger delay*) where there is a relevant delay between the time of activation of the respiratory muscle and the time of activation of the trigger (5, 27, 28).

*Double triggering* occurs when a sustained inspiratory effort persists beyond the inspiratory time of the ventilator, the cessation of inspiratory flow, or the onset of a mechanical expiration. This persistent inspiratory effort consequently triggers a second ventilator breath (5, 27, 28).

*Reverse triggering* occurs when ventilator insufflation triggers diaphragmatic muscle contractions by activating the patient's respiratory drive in response to a passive insufflation of the lungs (5, 27, 28).

Other asynchronies (called *cycle* or *termination asynchrony*) occur when there is a mismatch between the patient's neural inspiratory time and the ventilator's inspiratory time.

*Premature* or *short cycling* occurs when the neural inspiratory time is longer than the ventilator's inspiratory time. The patient's inspiratory effort continues, but the ventilator ends flow delivery. The premature cycling occurs at the beginning of expiratory phase (5, 27, 28).

*Auto-triggering* also known as *auto-cycling* occurs when a cycle delivered by the ventilator is not triggered by the patient (5, 27, 28).

*Prolonged* or *delayed cycling* occurs when the ventilator mechanical insufflation persists after the end of the neural inspiration or even during an active expiration (5, 27, 28).

## Built-In Software

Advances in technology resulted in new sophisticated ventilators equipped with built-in software, which may supply information about trends of patients on home ventilation. Built-in software data are potentially useful for the clinician to understand possible causes of not adequate ventilation (30). Companies are investing on software to provide data to clinicians to easily assess the quality of home ventilation. Data can be downloaded from a USB drive or device SD card or using wireless or Bluetooth communication. Data acquired from ventilators are mainly about adherence, leaks, and efficacy of therapy. Adherence data, expressed differently by different software, objectively establish non-use of ventilator and eventual causes of failure of ventilation. Moreover, air leaks could affect adherence creating discomfort, and this information is useful to eventually change settings and interface, if required. Furthermore, some modern ventilators provide data about efficacy of ventilation calculating the Apnea–Hypopnea Index, ventilation parameters, and cycling information during ventilator use (31, 32). Finally, data reports can include a section with detailed data analysis in which clinicians can analyze cycle by cycle the whole course of ventilation (32).

Although built-in software data are a useful tool for the clinician to understand home ventilation trends, there are several limitations on their routine use, such as lack of standardization of built-in software interpretation. In addition, there are no commercially available pediatric specific built-in software or validated data. Hence, currently, those data are available to understand trends of ventilation, but they cannot be considered as diagnostic tools.

As stated above, there are devices that provide only manual data reports (from a USB drive or device SD card) and others that offer in addition wireless/Bluetooth communication and, in few cases, smartphone-friendly data reports. Remote communication enables transmission of data using an internet connection (30). Thereby, clinicians can have easy access to ventilator data and can verify more frequently adherence, leaks, and efficacy of ventilation without the presence of the patient. A current limitation of telemonitoring systems is that data are not transmitted in real time and close follow-up is not possible. Another important limitation, shared by manual and remote data reports, is that, at this moment, information format provided vary greatly between different manufacturers, determining a non-homogeneous interpretation of data supplied to clinicians (29–31).

## Conclusions

A wide range of ventilators are currently available for clinical use. Bi-level and intermediate ventilators are mostly used for NIV. Simple or advanced CPAP (i.e., auto-titrating CPAP) devices are also available.

Volume-targeted and pressure-targeted (controlled) modes are available. Pressure-targeted (controlled) modes are preferable and flow-cycled modes such as PSV represent the most used NIV mode, as it is the most physiological mode.

The newest ventilators allow clinicians to use the hybrid modes, which combine the advantages of volume- and pressure-targeted (controlled) ventilation while limiting their drawbacks.

The use of in-built software may help clinicians to optimize the ventilator setting as well as check patient adherence to the treatment objectively.

## Author Contributions

MP contributed to the research and critical evaluation of the available literature and wrote the first draft of the paper. EV and AO contributed to the research and critical evaluation of the available literature and to writing sections of the paper. SC contributed to the research and critical evaluation of the available literature and paper. RC contributed to writing the first draft of the paper. All authors read and approved the final manuscript.

## Conflict of Interest

The authors declare that the research was conducted in the absence of any commercial or financial relationships that could be construed as a potential conflict of interest.
